# Impairment of adenosine signaling disrupts early embryo development: unveiling the underlying mechanisms

**DOI:** 10.3389/fphar.2023.1328398

**Published:** 2024-01-19

**Authors:** Talita Glaser, Patrícia Martins, Renata Beco, Carolina Adriane Bento, Angelica R. Cappellari, Sophia La Banca Oliveira, Christian Albert Merkel, Vanessa Fernandes Arnaud-Sampaio, Claudiana Lameu, Ana Maria Battastini, Henning Ulrich

**Affiliations:** ^1^ Department of Biochemistry, Institute of Chemistry, University of São Paulo, SãoPaulo, Brazil; ^2^ Department of Biochemistry, Federal University of Rio Grande do Sul, Porto Alegre, Brazil; ^3^ Department of Health (São Paulo—State), Medical School of the University of São Paulo (HCFMUSP), SãoPaulo, Brazil

**Keywords:** purinergic signaling, ERK1/2, calcium signaling, embryonic stem cells, adenosine, caffeine

## Abstract

Purinergic signaling has been implicated in many biological functions, including development. In this study, we investigate the functions of extracellular adenosine and adenosine receptors using a mouse embryonic stem cell (ESC) line and morula stages isolated from mouse embryos. Feeder-free mouse ESC was investigated in the absence and presence of the leukemia inhibitory factor (LIF), configuring undifferentiated cells and cells undergoing spontaneous differentiation. High alkaline phosphatase (ALPL) and low CD73 levels resulting in low adenosine (eADO) levels were characteristic for pluripotent cells in the presence of the LIF, while LIF deprivation resulted in augmented adenosine levels and reduced pluripotency marker expression, which indicated differentiation. Tracing ESC proliferation by BrdU labeling revealed that the inhibition of ALPL by levamisole resulted in a decrease in proliferation due to less eADO accumulation. Furthermore, caffeine and levamisole treatment, inhibiting adenosine receptor and eADO accumulation, respectively, reduced ESC migration, similar to that observed in the absence of the LIF. Pharmacological approaches of selective adenosine receptor subtype inhibition triggered specific adenosine receptor activities, thus triggering calcium or MAP kinase pathways leading to differentiation. In line with the *in vitro* data, mouse embryos at the morula stage were sensitive to treatments with A1 and A3 receptor antagonists, leading to the conclusion that A1 receptor and A3 receptor inhibition impairs proliferation and self-renewal and triggers inappropriate differentiation, respectively. The findings herein define the functions of eADO signaling in early development with implications for developmental disorders, in which adenosine receptors or ectonucleotidase dysfunctions are involved, and which could lead to malformations and miscarriages, due to exposure to caffeine.

## 1 Introduction

Regulation of adenosine 5′-triphosphate (ATP) and adenosine (ADO) concentrations in the extracellular space is a key factor in health and disease conditions (Segal et al., 1998; Burnstock, 2007; Di Virgilio and Adinolfi, 2017; Boison and Yegutkin, 2019). Described as extracellular messengers first by Burnstock in the 1970s (Burnstock et al., 1970; Burnstock et al., 2011), nucleotides, nucleosides, and their derivatives earned great attention as fine-tuning, widespread, and primitive cell signalers (Knight, 2010).

Several ATP-consuming and ATP-generating pathways coexist in a tightly orchestrated network in order to modulate extracellular concentrations of purinergic components (Zimmermann et al., 2012). ATP is released from cells in a controlled manner, mediated by vesicular exocytosis, connexin/pannexin hemichannels, and transporters, or in an uncontrolled manner whenever membrane integrity is compromised (Lohman et al., 2012). Membrane-anchored ectoenzymes convert ATP into ADP and AMP and, posteriorly, into adenosine (Verkhratsky et al., 2020; Giuliani et al., 2021). Among those enzymes, the NTPDase family comprises diphosphohydrolases that hydrolyze tri- or diphosphate nucleotides, ultimately generating AMP. The NPP family comprises pyrophosphatase or phosphodiesterase enzymes, which, besides catalyzing the same breakdown steps as NTPDases, also produce AMP directly from ATP. CD73, an ecto-5′-nucleotidase, metabolizes the AMP into adenosine, and thus, alkaline phosphatase (ALPL) is able to catalyze all the steps for generating adenosine; however, its efficiency of generating adenosine from AMP is lower (Braun et al., 2000; Robson et al., 2006; Boison and Yegutkin, 2019). In addition to the classical route of extracellular ATP breakdown until the generation of ADO, alternative routes using cAMP and NAD^+^ as substrates also produce adenosine (Linden et al., 2016; Puelles et al., 2020). Contrarily, enzymes such as adenylate kinase and nucleoside diphosphate kinase can regenerate ATP (Zimmermann et al., 2012), and adenosine levels can be reduced by its direct breakdown of adenosine into inosine by adenosine deaminase (ADA) or by recapture into the cell through nucleoside transporters (Boison and Yegutkin, 2019).

While ATP exerts its extracellular effects through P2 receptors, either ionotropic (P2X1-7) or metabotropic (P2Y1-14), adenosine activates G-coupled P1 receptors, namely, A1, A2A, A2B, and A3 receptors (Burnstock, 2007), with the A1 and A2A receptors being the ones with the highest binding affinities in rats, i.e., with the lowest *Ki* values. In humans, adenosine is nearly equipotent in activating A1, A2A, and A3 receptors (Fredholm et al., 2011). The triggered intracellular effects depend on the respective type of activated G protein: A1 and A3 receptors are usually coupled to G_i_ proteins, inhibiting cAMP synthesis; in contrast, A2A and A2B receptors are commonly coupled to G_s_ proteins, thus stimulating cAMP production (Jacobson and Gao, 2006). Generally, adenosine is an environmental cue related to hypoxia and stress and exerts mostly protective and immunosuppressive roles, counteracting pro-inflammatory roles triggered by extracellular ATP (eATP), e.g., through the P2X7 receptor (Arnaud-Sampaio et al., 2020).

Purinergic signaling is present in the early stages of embryonic development in a wide variety of organisms and has central roles in the formation of many organs (Burnstock and Ulrich, 2011). In mammals, the totipotent stem cells originated through egg fertilization give rise to pluripotent stem cells following blastocyst formation, and cell commitment further increases with lineage specification, generating multipotent cells, such as neural stem cells (NSCs) or mesenchymal stem cells (MSCs) (Plusa and Hadjantonakis, 2014; Cavaliere et al., 2015). Diverse cues interact to determine whether cells will divide symmetrically or asymmetrically or remain quiescent or senescent. The balance between extracellular ATP and ADO levels may shift or undermine proliferation, migration, and differentiation events, controlling cell fate (Ulrich et al., 2012; Cavaliere et al., 2015). During mammalian embryonic development, ATP promotes the increase in intracellular Ca^2+^ concentration through P2 receptors (Kubo, 1991; Nakano et al., 2003), possibly mediating cell proliferation and differentiation events (Burnstock and Ulrich, 2011). On the other hand, ADO at a micromolar range impairs the normal cleavage of *in vitro*-developing mouse embryos (Nureddin et al., 1990), and several alterations in the expression patterns of nucleotide- and nucleoside-metabolizing enzymes have been described throughout embryonic development (Nabinger et al., 2020).

In MSCs, ADO receptors seem to have pro-osteogenic and immunomodulatory roles (Cavaliere et al., 2015). In NSCs, although A1, A2A, and A2B P1 receptors have been detected (Stafford et al., 2007), as well as NTPDases and ALPL, their roles are poorly described; it is also not clear which effects are mediated by ADO signaling, with the P2 receptors being the ones with better-characterized roles in neurogenesis (Resende et al., 2007; Glaser et al., 2014; Cavaliere et al., 2015; Ribeiro et al., 2019).

Given the understudied role of ADO signaling and metabolism in stem cell function and considering that the dysfunction of purinergic signaling disrupts normal development in animal models (Burnstock and Ulrich, 2011), understanding the roles of ectonucleotidases and P1 receptors in stem cell proliferation and differentiation is highly relevant.

Caffeine exposure during pregnancy is closely related to some health outcomes, such as increased cardiovascular mortality and abortion of normal karyotype fetuses (Signorello et al., 2001). Coffee consumption at early stages of development may impair oviductal embryo transport, embryonic development, and uterine receptivity (Qian et al., 2018). Caffeine antagonizes all types of adenosine receptors (A1, A2A, A2B, and A3 subtypes) (Rivera-Oliver and Díaz-Ríos, 2014). The roles of these receptors during prenatal stages still need to be elucidated.

For that reason, we studied the roles of ADO receptors and ectonucleotidases in the stemness of mouse embryonic stem cells (mESCs) maintained by the presence of leukemia inhibitory factor (LIF) and the effects of its withdrawal when the ADO system is altered. Importantly, we found that ALPL and ADA favor a lower concentration of extracellular ADO, controlling ESC proliferation through A1 receptor activation, triggering intracellular calcium concentration [Ca^2+^]_i_ transients and self-renewal, while CD73 favors high concentrations of extracellular ADO that binds to the A2B and A3 receptors, which have a lower affinity to ADO, triggering the MAP kinase pathway and leading to the differentiation of mESCs *in vitro* and *in vivo*. Caffeine and subtype-selective ADO receptor antagonists were used to probe their effects on stem cell proliferation and differentiation.

## 2 Methods

### 2.1 Culture of mouse embryonic stem cells

In our study, we used the E14Tg2A mESC line, which does not need a feeder layer to grow. ESCs were expanded and maintained in a culture medium containing DMEM high, 15% fetal bovine serum (FBS), 1% nonessential amino acids, 2 mM L-glutamine, 50 μg/mL of penicillin and streptomycin, 2 mM sodium pyruvate, and 10^3^U/ml ESGRO LIF (Merck Millipore), which maintains the cells in an undifferentiated state by the STAT-3 activation process that has been well reviewed by Chambers and Smith (2004). Cells were passed when they had reached 80% of confluence. The ESC received two different treatments, with the LIF (undifferentiated cells) and without the LIF (differentiated cells), for 96 h.

### 2.2 Flow cytometry assays (phenotyping)

In order to investigate the role of adenosine receptors in pluripotency, we cultivated and treated ESC with the inhibitors of ALPL (1 mM levamisole), CD73 (α,β-methyleneadenosine 5′-diphosphate sodium salt), and antagonists of the A1R (10 nM PSB 36), A2AR (645 nM ANR 94), A2BR (10 nM PSB 603), A3R (50 nM MRS 3777), and the less specific inhibitor, caffeine (50 μM). After 96 h, the cells were washed, trypsinized, and fixed with a 4% paraformaldehyde solution. The cells were washed in phosphate-buffered saline (PBS) and blocked with a 4% normal donkey serum and 0.1% Triton-X in PBS for 60 min. The cells were then incubated with mouse anti-SSEA1 antibody (Millipore, 1:500 dilution) for 2 h. In sequence, the cells were washed with a 4% normal donkey serum and 0.1% triton-x in PBS and incubated with Alexa Fluor 555 secondary antibody (Life Technologies, 1:500) for 1 h. As the negative control, we used the cells that had undergone the whole process but were not labeled by the primary antibodies. The percentage of marked cells was measured in the Attune^®^ flow cytometer (Thermo Fisher Scientific). At least 50,000 events were acquired per sample, and the analysis was performed in FlowJo software.

### 2.3 RT PCR-qPCR

Total RNA was extracted from undifferentiated and differentiated ESCs using Trizol (Life Technologies) according to the manufacturer’s instructions. RNA integrity was evaluated by separation in 1% agarose gel. cDNA was synthesized with 2 μg of RNA in a total volume of 20 μL in the presence of oligo dT 10nm, 10 mM dNTP, RiboLock, 200 U/ml RevertAidTM H Minus Moloney Murine Leukemia Virus-antisense transcriptase (MMMLV, Fermentas Inc., Hanover, MD), and MilliQ water for 60 min at 42°C. The cDNA was amplified with the StepOne Real-Time PCR System (Thermo Fisher Scientific). The reaction was performed in 15 μL of buffer reaction containing 2 µg cDNA, 7.5 μL of SYBR Green master mix (Life Technologies), and 0.3 μL of each sequence-specific pair of primers (Table 1). The initial denaturation step was performed for 10 min at 95°C, followed by 50 cycles for 15 s at 95°C and annealing for 1 min at 60°C. The quantification of gene expression was analyzed as a relative value compared to an internal reference, glyceraldehyde-3-phosphate dehydrogenase (GAPDH), whose expression levels did not vary between the experimental conditions.

**TABLE 1:** Primer sequences for RT–qPCR.

Gene	Forward 5′-3′	Reverse 5′-3′	Amplicon (bp)
ENTPD1	CAC​AGG​GGT​GGT​GCA​GCA​GT	TCG​GCC​AGG​TAC​GCA​CCG​AT	99
ENTPD2	CGG​GCT​CCT​GCT​ACT​GTG​CG	GCC​AGC​ATC​CAG​AAC​GAT​GCC​A	82
ENTPD3	CCA​ACC​CGC​AGC​GAA​AGC​AG	TCC​TCG​AGA​GGG​CCC​TGA​AGC	100
ENTPD5	TTC​TGC​TTT​TGT​GCA​TCA​GC	CTT​CTG​GGG​ATC​GAG​TCT​TT	84
ENTPD6	CGG​CTG​GCT​ATC​TTG​GGC​GG	CGG​GGA​GAC​AGA​CAG​GGG​CT	80
ENTPD8	TGG​TGA​AGG​CAA​TCA​ATG​TT	GCC​ACT​GGT​ACA​CAA​ACA​GG	99
ENPP1	CCT​GCT​GCT​CGG​TTG​AGA​CCC	GCT​GGT​TTG​GCT​CCC​GGC​AA	100
ENPP2	GGT​GGG​GAG​GCC​AAC​CGC​TA	TCT​CCG​CTC​GTG​AGG​GAT​GCT	95
ENPP3	GGG​GCT​GGG​ACT​GGG​ACT​CA	CGG​CAG​CCC​TCC​AGT​CCT​CT	93
E5NT/CD73	AGG​TGT​GGA​CAT​CGT​GGT​GGG​A	GGG​TAC​TTC​CCC​GCA​CGC​AC	90
ALPL	CCT​GCG​CTG​GGC​CAA​GGA​TG	GGC​CGA​GTG​TGC​GTA​GCC​TG	97
ADA	ACA​AGC​CCC​TCT​CGC​TCC​CA	TGA​TGG​CCT​CTC​TGC​AGC​CC	81
A1	GCA​GGA​AAG​GGG​TGG​ATG​AA	CGC​TGG​TGC​CCG​ATT​CTT​A	115
A2A	TCC​TCA​CGC​AGA​GTT​CCA​TC	TAC​CCG​TCA​CCA​AGC​CAT​TG	99
A2B	GGA​ACC​GAG​ACT​TCC​GCT​AC	GAC​TGA​GAG​TAG​ACT​GCG​CC	111
A3	GAA​GTA​AGA​ACG​GTG​GCC​CT	CCC​TGC​CTT​CCC​ATT​AAC​CT	72
OCT3/4	ATG​CCG​TGA​AGT​TGG​AGA​AG	TGT​ACC​CCA​AGG​TGA​TCC​TC	123
NANOG	CAG​AAA​AAC​CAG​TGG​TTG​AA	GCA​ATG​GAT​GCT​GGG​ATA​CT	81
GAPDH	AGC​TTC​GGC​ACA​TAT​TTC​ATC​TG	CGT​TCA​CTC​CCA​TGA​CAA​ACA	89

### 2.4 BrdU incorporation assay

In order to investigate the role of adenosine receptors and also the ALPL and CD73’s role in cell proliferation and cell cycle, a BrdU incorporation assay was performed. To analyze the adenosine receptor’s function, ESC were cultivated and treated with the inhibitors and antagonists of the A1R (10 nM PSB 36), A2AR (645 nM ANR 94), A2BR (10 nM PSB 603), A3R (50 nM MRS 3777), and 50 μM caffeine (less specific inhibitor). All treatments were carried out in the presence of the LIF and adenosine (P1 receptor agonist) for a total of 96 h. For ALPL and CD73 function analysis, ESCs were treated with the respective inhibitor of each enzyme, 1 mM levamisole and 5 μM adenosine 5′-(α,β-methylene)diphosphate, both in the presence of the LIF. Cells cultivated in the presence and absence of the LIF were used as the control groups. After 96 h, cells were incubated with 0.2 μM 5-bromo-2-deoxyuridine (BrdU) for 1 h. Cells were then washed, trypsinized, and fixed with 75% ethanol. Next, ESCs were washed with PBS and incubated for 30 min in a 1.5 M HCl solution and neutralized with 0.1 M sodium tetraborate. In sequence, cells were washed three times with PBS and blocked with a 2% fetal bovine serum, 0.1% Triton-X solution in PBS, for 60 min. After washing with PBS, they were incubated in mouse anti-BrdU antibody (Millipore, 1:100 dilution) for 1 h. Afterward, cells were washed with a 4% fetal bovine serum, 0.1% Triton-X solution in PBS and incubated with Alexa Fluor 488 secondary antibody (Life Technologies, 1:1,000 dilution) for 1 h. Cells that had undergone the whole process but were not marked with the primary antibodies were used as the negative control. Percentages of BrdU-positive cells were measured in an Attune^®^ flow cytometer (Applied Biosystems). At least 50,000 events were acquired per sample and further analyzed with the FlowJo software.

### 2.5 Ectonucleotidase enzymatic activity assay

Undifferentiated cells were cultivated and treated in the presence or absence of the LIF. After 48 h of treatment, the culture medium was replaced and 500 µL of cells in the culture medium with or without the LIF were plated in a 24-well plate (*cell plus* treated for adherence agar-free). The incubation medium contained 5 mM KCl, 120 mM NaCl, 10 mM glucose, and 20 mM Hepes Buffer (pH 7,4). Substrates containing phosphate used were 100 µM ATP, 100 µM ADP, and 100 µM AMP. When ATP or ADP were used, the incubation medium was supplemented with 2 mM CaCl_2_; if AMP was used, 2 mM MgCl_2_ was added to the incubation medium.

At first, the wells were washed 3x with the phosphate-free incubation medium at 37°C. Cell incubation started with the addition of 300 µL of incubation medium enriched with the substrate/well. 150 μL aliquots were collected after 0, 5, 10, 20, 30, 60, and 90 min. An extra aliquot of phosphate-free incubation medium was collected in order to quantify the basal nucleotide levels secreted by these cells. In addition, an aliquot of the incubation medium was collected to obtain the nucleotide spontaneously hydrolyzed at the same different times of incubation. All aliquots were kept on ice (–4°C). The collected supernatants were centrifuged for 30 min at 10.000 x g at 4°C.

### 2.6 Nucleotide and nucleoside content analysis by high-performance liquid chromatography

After the enzymatic assay, 20 μL aliquots were analyzed using the HPLC method (Supelcosil LC-18, 25 cm 64.6 mm, Supelco) in a Shimadzu liquid chromatograph (Shimadzu, Japan). Through a linear gradient (first, using a 100% buffer solution named A containing 150 nmol/L PBS and 150mM KCl at pH 6 and, second, using a 100% buffer solution named B containing 15% C_2_H_3_N in buffer solution A), molecules were separated. To quantify nucleotides and their subproducts, a 254 nm absorbance wavelength was applied. All peaks were identified by their retention time and quantity and compared against their respective standards. Purine concentration is expressed as nmol of the released product (mean ± S.D.). All experiments were run in triplicates to discount the effect of non-enzymatic nucleotide hydrolysis. When quantifying nucleotides, aliquots containing the incubation medium with nucleotides and without cells were used to standardize the experiment.

### 2.7 Determination of total proteins

After aliquot collection from cell plates, the residual incubation medium was removed. Cells that remained attached to the plate bottom were suspended in 1 M NaOH, and total protein concentration was determined through Bradford’s method using Bovine serum albumin (BSA) (2 mg/mL) as the standard protein.

### 2.8 [Ca^2+^]_i_ imaging

Undifferentiated and differentiated E14Tg2A ESCs were loaded with 5 µM of Fluo3-AM for 45 min at 37°C in DMEM high glucose in 0.5% Me2SO and 0.06% of the nonionic surfactant pluronic acid F-127 (Sigma-Aldrich). After loading with Fluo-3 AM, the cells were incubated with extracellular buffer (140 mM NaCl, 3 mM KCl, 1 mM MgCl2, 2 mM CaCl2, 10 mM HEPES, and 10 mM glucose at pH 7.4) (Glaser et al., 2020). Ca^2+^ imaging was performed using the Inverted Research Microscope ECLIPSE-TiS (Nikon, Melville, NY) equipped with a 14-bit high-resolution CoolSNAP HQ2 CCD camera (Photometrics, Tucson, AZ) and analyzed with the NISElement software (Nikon) using image acquisition rates of two frames per second. Fluo-3 fluorescence was excited with a xenon lamp at 488 nm, and the emitted light was detected using a bandpass filter at 515–530 nm. Intracellular calcium flux was monitored in cells stimulated with adenosine (2 µM).

### 2.9 [Ca^2+^]_i_ measurements by microfluorimetry

Undifferentiated cells were plated in a 96-well plate with 100 μL of culture medium per well. They were incubated for 60 min at 37°C with the FlexStation Calcium Assay kit (Molecular Devices Corp.) containing 2.5 mM probenecid at a final volume of 200 μL per well. Fluorescence of samples was excited at 485 nm, and fluorescence emission was detected at 525 nm. The basal fluorescence intensity for [Ca^2+^]_i_ was measured in unstimulated cells, and then, the induced [Ca^2+^]_i_ transients were measured in cells stimulated with adenosine (agonist) and a P1 inhibitor cocktail for each P1 receptor subtype (Table 2). The responses to adenosine and P1 inhibitor cocktail addition were determined by the fluorescence peak minus the basal fluorescence intensity using SoftMax2Pro software (Molecular Devices Corp.). The data were expressed as the mean values ± standard errors.

**TABLE 2:** Cocktails of P1 subtype antagonists. X indicates that the drug was used in the mix.

	Drugs applied				
Active receptor	ADO	PSB36	ANR94	PSB603	MRS3777
	2 μM	10 nM	645 nM	10 nM	50 nM
A1	X	-	X	X	X
A2A	X	X	-	X	X
A2B	X	X	X	-	X
A3	X	X	X	X	-

### 2.10 Western blotting

Undifferentiated and differentiated E14Tg2A cells were stimulated for different times (2 µM adenosine for 30 s, 1, 2, 5, and 10 min) in order to evaluate the most suitable time of stimulation. The cells were then washed with PBS, released with TBS + EDTA solution, and homogenized in a lysis buffer (10 mM Tris-HCl, 150 mM NaCl, 20 mM EDTA, 1% Triton X-100, and 8 M urea; pH 7.5) plus protease inhibitor. Thirty micrograms of protein were separated by SDS-PAGE on a polyacrylamide 10% gel at a constant voltage of 100 V for 1 h and then transferred onto a nitrocellulose membrane in a wet system for 1 h at the constant amperage of 400 mA. The membranes were washed with TBS and then incubated with 5% BSA in TBS-Tween for 60 min in order to block nonspecific binding. Next, the membranes were incubated with primary antibodies for p-ERK 1/2 (Cell Signaling, 1:500), ERK 1/2 (Cell Signaling, 1:1,000), ALPL (Thermo Fisher Scientific, 1:200), CD73 (Sigma-Aldrich, 1:2,000 dilution), and actin (Sigma-Aldrich, 1:1,000 dilution) overnight at 4°C. The membranes were washed three times with TBS-T and then incubated with Alexa Fluor 488 and 647 secondary antibodies (Life Technologies, 1:1,000 dilution) for 1 h under agitation. The membranes were washed again and scanned with Typhoon (GE Healthcare Life Sciences). The resulting bands were quantified in the ImageJ software. After the optimal adenosine concentration and time of stimulation had been defined, a new experiment was conducted with undifferentiated and differentiated cells stimulated with adenosine and, shortly thereafter, inhibited with a P1 receptor inhibitors cocktail, which kept only one adenosine receptor subtype active at the time (Table 2). The doses were doubled from the EC50 recommended by the manufacturer (Tocris). Finally, proteins of the cells were extracted and had their expression quantified according to the same protocol described above.

### 2.11 *In vivo* procedures

One intraperitoneal injection of gonadotropin (from pregnant horse: 5IU/animal) was administered to female C57BL/6 mice, and after 48 h, they were injected with human chorionic gonadotropin (hCG: 5 IU/animal). Then, they were mated with the same lineage males. The embryos at 3 days after coitus were collected by washing the infundibulum with M2 medium (Merck Millipore). The embryos were isolated by 10 min of centrifugation at 12,000 *g* at 4°C (Chambers and Smith, 2004). Acidic Tyrode’s solution (pH 2–5 + 0.04% polyvinylpyrolidone) removed the zona pellucida by a 15–30 s incubation at 36°C. An adherence recovery step of 1 h in PBS +10% FCS at 36°C was followed. Then, the embryos were transferred to 35 mm Petri dishes containing microdroplets of M2 medium for 10 min at 37°C, followed by embedding with KSOM medium (Merck Millipore) and mineral oil coverage. They were kept in an incubator at 36°C and 5% CO_2_, 5% O_2_, and 90% N_2_, and the medium was changed every 24–36 h. When they reached the morula/blastocyst stage, we applied the following for 2 days: 10 nM PSB36 (A1 receptor antagonist) or 50 nM MRS 377 (A3 receptor antagonist). We used 15 embryos for each condition plus the experimental control.

### 2.12 Immunofluorescence assay

Embryos were prepared following the work of Dehghani et al. (2005). In brief, after the treatment incubation with PSB36 or MRS377, the embryos were fixed with 2% PFA in PBS for 30 min at room temperature. Then they were permeabilized by incubating with 0.5% Triton X-100 for 10 min, followed by three washing steps with PBS supplemented with 5 mM glycine and 0.1% Tween for 10 min each. The embryos were equilibrated with an intracellular buffer for 5 min (100 mM KCl, 5 mM MgCl2, 3 mM EGTA, and 1% BSA in 20 mM HEPES; pH 6.8).

Anti-SSEA-1 antibody was incubated with the embryos, following the manufacturer’s instructions, in the intracellular buffer at 4°C for 48 h inside a humid chamber. The embryos were rinsed twice with the intracellular buffer and incubated with anti-mouse IgG conjugated with Alexa Fluor 545 for 2 hs. After secondary antibody staining, the embryos were washed twice with the intracellular buffer and incubated in DAPI (1 mg/mL) for 10 min. Slides were mounted with Fluoromount-G. Images were acquired with a Nikon epifluorescence microscope coupled with a CCD camera.

### 2.13 Wound healing

Mouse embryonic stem cells (E14Tg2A) were plated on gelatin-coated (0.1% porcine skin gelatin, Sigma-Aldrich) plastic dishes (24 wells plate, Sarstedt). With a p20 pipette tip, a straight scratch was designed when the confluence had reached 70% (24 hs). Then, the culture medium was changed in the presence of the adenosine receptor antagonists. The cultures were scanned using the TissueFAXS Q cytometer (TissueGnostics) every 12 h for 3 days. The areas of the scratch were automatically measured with the StrataQuest software (TissueGnostics). The variation of the area was calculated by subtracting the area of the last picture from the area of the first one (Δ area).

### 2.14 Statistical analysis

The results were statistically analyzed with ANOVA, and a *t*-test and multiple comparisons between groups were performed using the Bonferroni *post hoc* test*.* All analyses were carried out using GraphPad Prism 8.0 software (GraphPad Software Inc., San Diego, CA), and the criteria for statistical significance were set at *p* ≤ 0.05.

## 3 Results

### 3.1 Ectoenzymes and pluripotency

Embryonic stem cells comprise the inner cell mass of the blastocyst, and their *in vitro* differentiation resembles the early stages of embryonic development. We used this model to study the roles of adenosine receptors and ectonucleotidases in the stemness maintained by the presence of the LIF and the effects of its withdrawal (Grosso et al., 2017; Poole et al., 2017).

**FIGURE 1:**
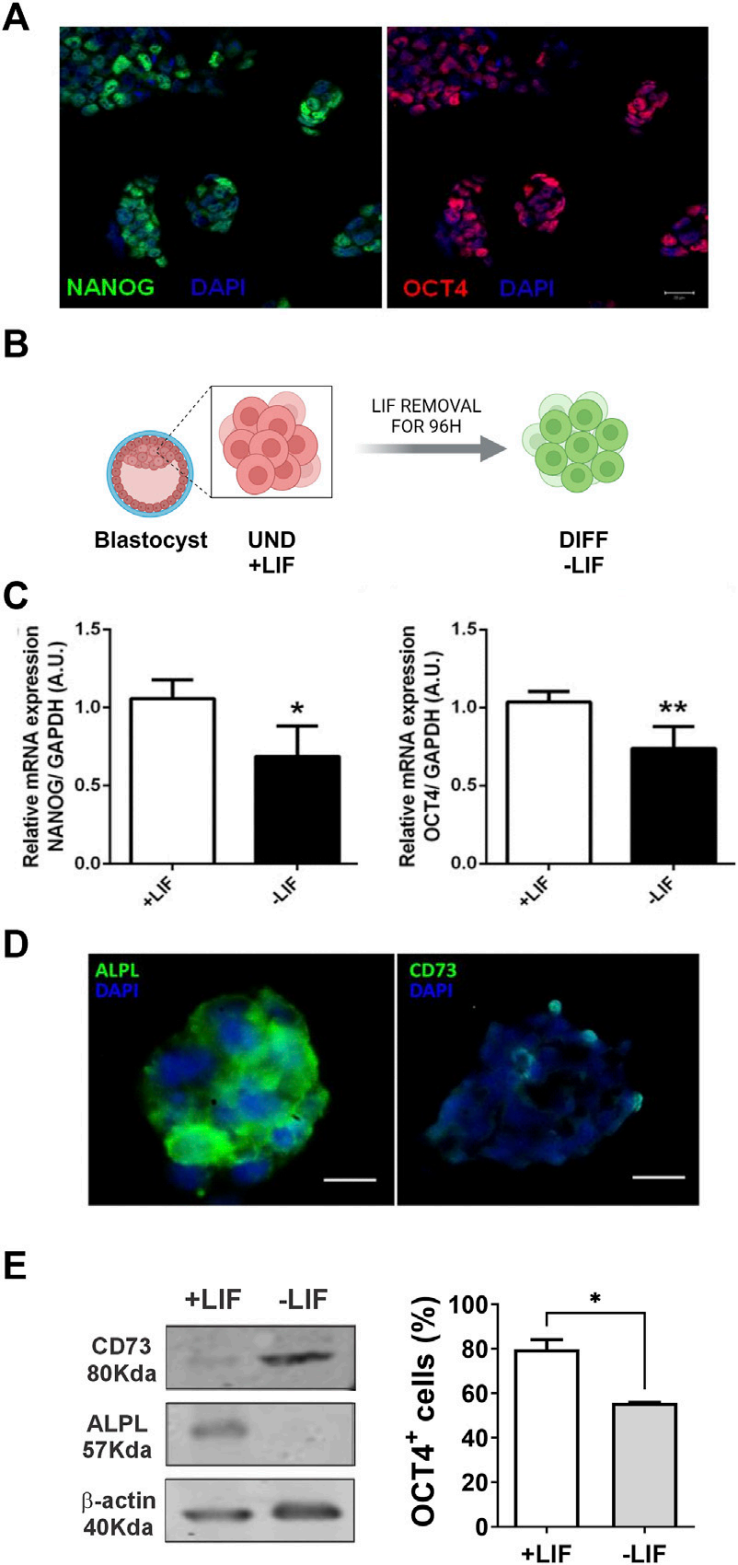
Expression of purinergic system components in E14Tg2A mESC: immunofluorescence studies of embryonic stem cell colonies. Cells were immunolabeled for **(A)** pluripotency markers (OCT4 in red and NANOG in green) and **(D)** ALPL and CD73 enzymes (in green); the nuclei are in blue (DAPI). Scale bar = 25 μm. **(B)** Experimental design showing that cells with LIF are undifferentiated and those under 96 hs depletion of LIF are spontaneously differentiated. **(C)** Pluripotency marker, OCT4 and NANOG, expression by real-time PCR with cells cultured in the presence and absence of the LIF. **(E)** As indicated by Western blotting analysis (left panel), the removal of the LIF resulted in reduced ALPL expression and increased CD73. Flow cytometry (Right panel) was carried out for OCT4 detection cells cultured in the presence and absence of the LIF. One-way ANOVA was conducted. *p ≤ 0.05. **p ≤ 0.01. UND, undifferentiated; DIFF, differentiated. The experiments were independently repeated at least three times in triplicates. Created with BioRender.com.

The expression of the transcription factors NANOG and OCT4 evidence undifferentiated pluripotent cells. During development, the levels of NANOG and OCT4 decay as the cells fall in the differentiation process to generate other cells of the embryo (Glaser et al., 2014). This step is crucial for embryonic development as failure in this step may lead to early miscarriage. As expected, we observed a significant decrease of both NANOG and OCT4 in the cells after 96 h of LIF deprivation (Figures 1A–C).

In view of that, we investigated the presence of ALPL and CD73, both ectoenzymes that are able to convert AMP in ADO. Interestingly, mESCs under undifferentiated conditions expressed high levels of ALPL, as previously described (Tsuji et al., 2008), while the CD73 level was low (Figure 1D). Once the cells are kept in LIF deprivation, ALPL protein levels decrease substantially, as indicated by Western blot, while CD73 levels increased (Figure 1E).

**FIGURE 2:**
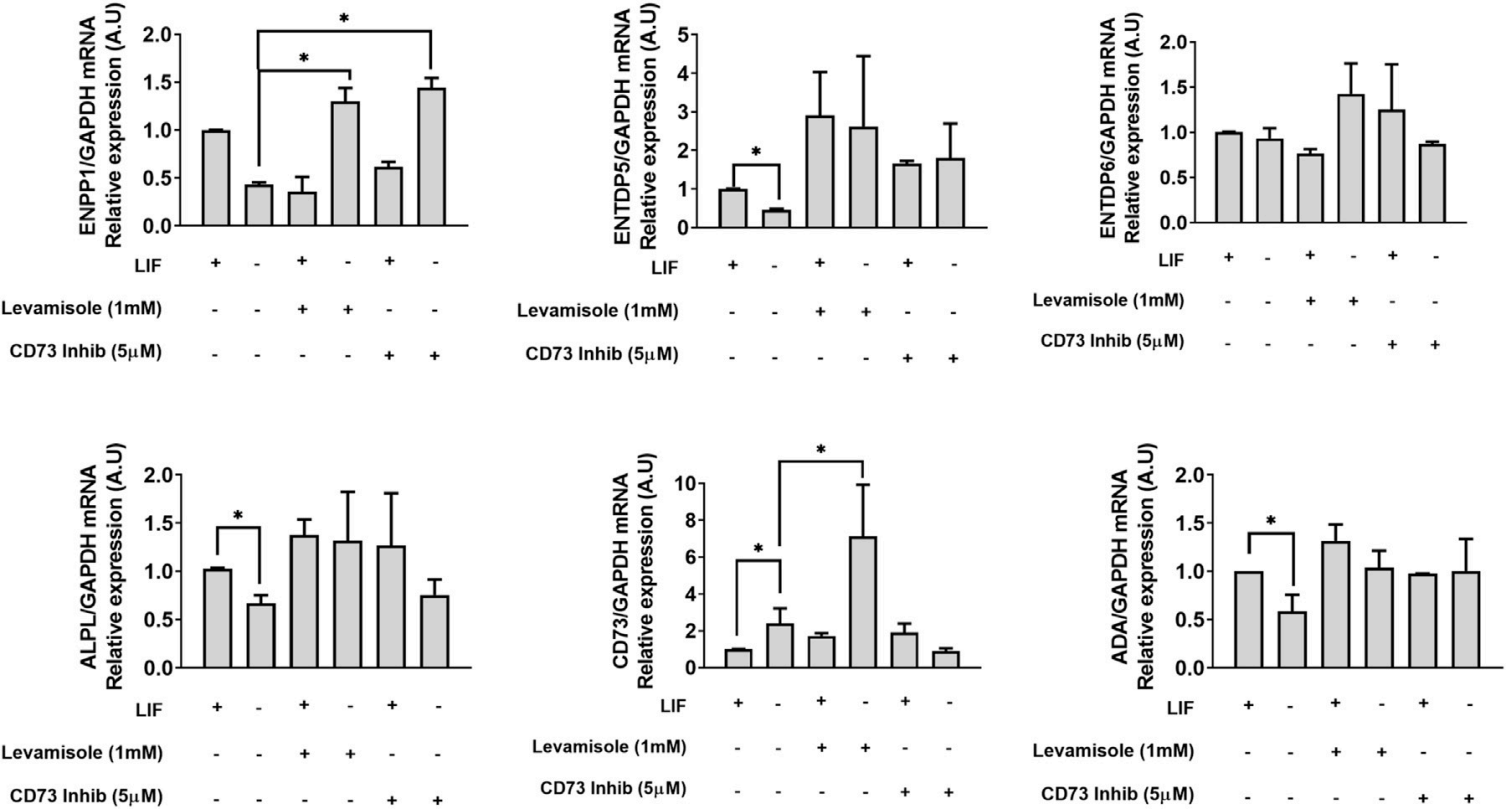
Ectonucleotidase and adenosine deaminase expression in the absence and presence of the LIF and inhibition of CD73 and alkaline phosphatase. mESCs were treated for 96 h under the indicated conditions and assayed by real-time RT–PCR for ENPP1, 5,6, alkaline phosphatase, CD73, and adenosine deaminase (ADA) gene expression. Relative expression levels were normalized to GAPDH expression levels, which did not change under the used experimental conditions. One-way ANOVA was conducted followed by the Bonferroni post-hoc test; *p ≤ 0.05. The experiments were independently repeated at least three times in triplicates.

Considering that the extracellular levels of the activators (ATP, ADP, and ADO) of the purinergic system are controlled by ectoenzymes, we performed RT–PCR experiments to evaluate the presence of the different enzymes. The detected ones were ENPP1, ENPP2, ENPP3, ENTDP1, ENTDP2, ENTDP3, ENTDP5, and ENTDP6, the ones with differential expression are plotted in Figure 2.

As enzyme pathways are synchronized, we hypothesized that the activity modulation of ALPL or CD73 would change the expression pattern of some of the enzymes. We observed that the only enzyme affected at the expression level by the treatment with an ALPL inhibitor (levamisole) was CD73 (Figure 2). In this case, levamisole induced a six-fold increase in the mRNA levels of CD73. These data indicate a feedback response between CD73 and ALPL expression, corroborating the data of inverse expression levels of the enzymes, probably for the maintenance of the steady extracellular levels of ADO for each development step. We could not observe any change upon CD73 inhibition with α,β-methyleneadenosine 5′-diphosphate sodium salt (α,β-MeADP). ENPP1 showed a three-fold increased expression under the inhibition of CD73 or ALPL (Figure 2), suggesting it to be an important checkpoint for the availability of the substrate (AMP) for enzymatic activity. In addition, adenosine deaminase (ADA), which converts ADO in inosine, was highly expressed in the undifferentiated cells *versus* the LIF-deprived ones; however, the expression levels were insensitive to both ALPL and CD73 inhibition (Figure 2).

**FIGURE 3:**
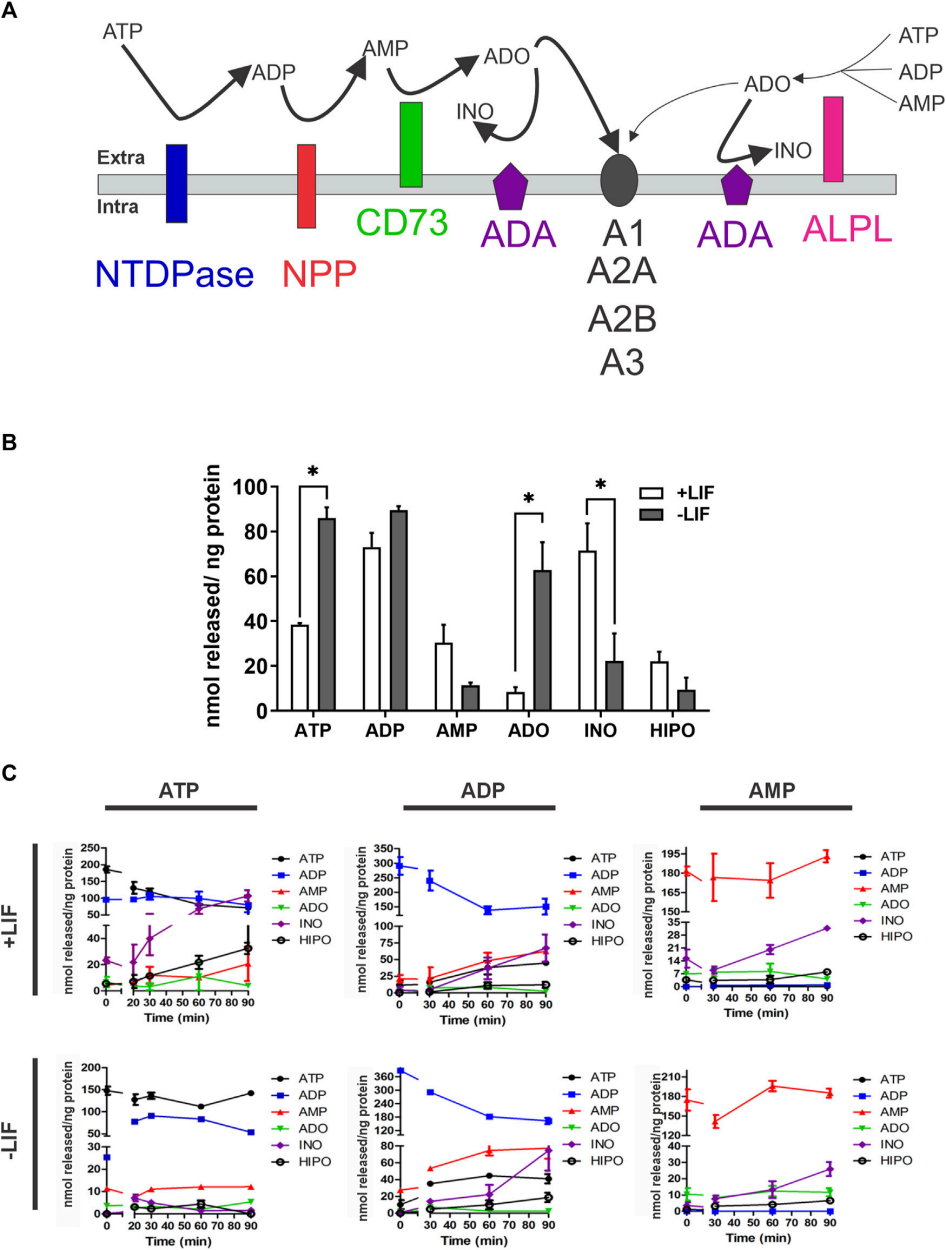
Extracellular nucleotide and nucleoside concentrations obtained by HPLC after enzymatic assay in undifferentiated (+LIF) and differentiated (-LIF) mouse ESC **(A)**. Illustration of the pathway of nucleotide conversion by ectonucleotidases. CD73 is more efficient in generating ADO, while ALPL can use different substrates. ADO activates P1 receptors (A1, A2A, A2B, and A3), which have distinct sensitivity **(B)**. Extracellular basal concentration of tri-, di-, and monophosphate nucleotides (ATP, ADP, and AMP) and nucleosides (adenosine-ADO, inosine-INO, and hypoxanthine-HIPO) upon 90 min incubation in a phosphate-free buffer. Experiments were repeated at least three times in triplicates. A one-way ANOVA test was conducted followed by the Bonferroni post-test with *p* ≤ 0.05 **(C)**. Extracellular nucleotide and nucleoside concentrations in the extracellular medium during enzymatic assay. The cells were incubated with a phosphate-free buffer containing ATP (100 mM), ADP (80 mM), or AMP (80 mM), and the supernatant collected at different time points (0, 5, 10, 20, 30, 60, and 90 min) in triplicates. Experiments were repeated at least three times. Data were collected from three independent experiments in triplicates.

The modulation of enzymatic activity is a common and efficient cellular strategy to balance pathways and cell response. Any alteration in the balance of the described purinergic pathway may disrupt the signaling or induce new cellular responses (Figure 3A). Therefore, we evaluated the extracellular levels of ATP, ADP, AMP, ADO, inosine, and hypoxanthine in the medium of undifferentiated (+LIF) and differentiated cells (-LIF) (Figure 3). We observed a significant shift of extracellular ADO concentration in differentiated cells (average of 520 nmol/ng protein) when compared to undifferentiated ones (average of 2 nmol/ng) (Figure 3B), which corroborates our results of increased CD73 expression, which is needed for efficient ADO production and lower ADA expression.

Moreover, we performed an enzyme activity assay to understand the differences in the process of nucleotide hydrolysis using ATP, ADP, and AMP as substrates over time in undifferentiated (the absence of the LIF) *versus* differentiating cells (the presence of the LIF) (Figure 3C). The levels of substrate formation were similar in both conditions, except for inosine, which was higher in undifferentiated cells when ATP was used as the substrate. The data confirm our findings of ADA being highly expressed and functional in undifferentiated cells, leading to lower levels of ADO due to higher consumption by ADA.

**FIGURE 4:**
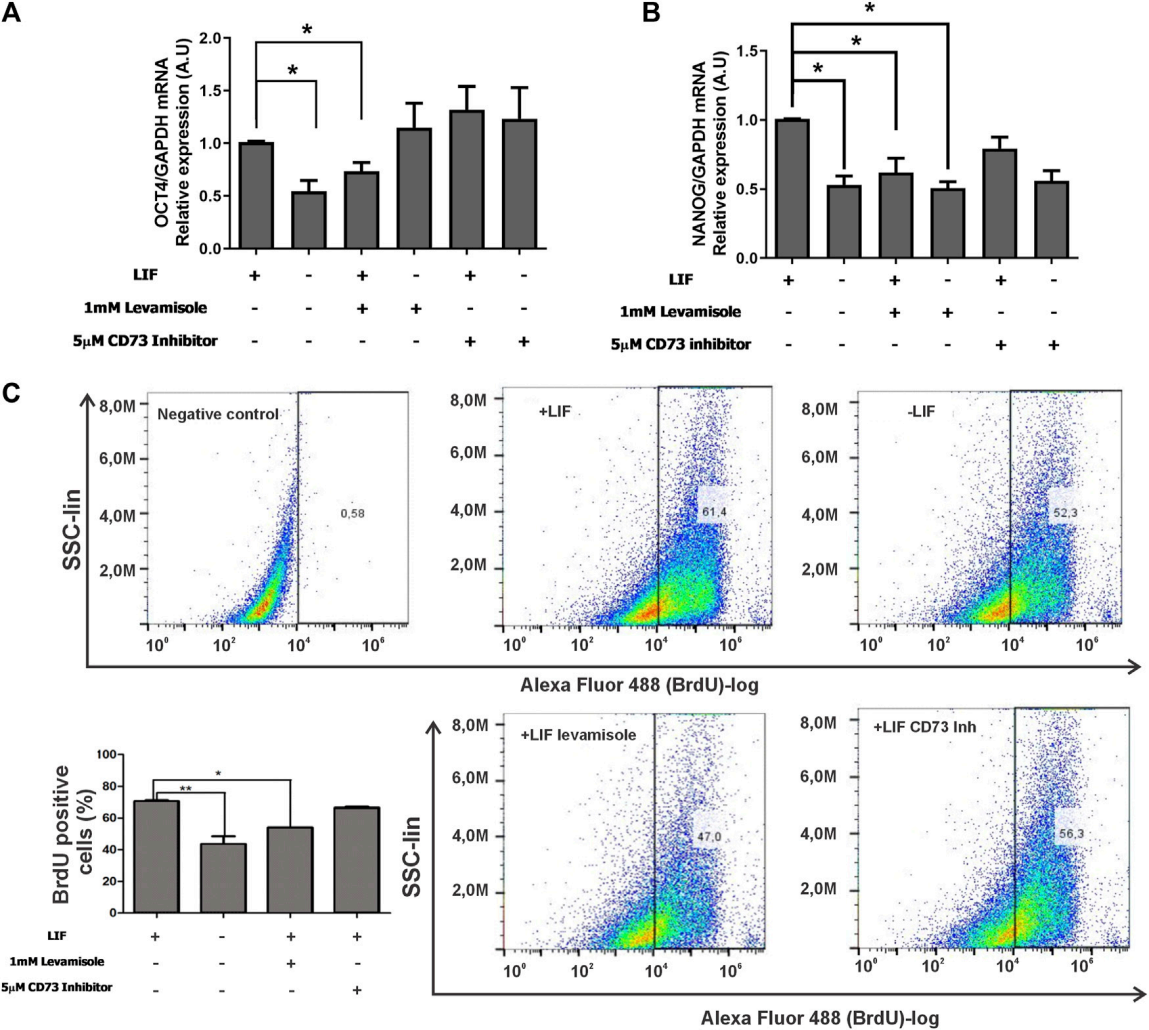
ALPL and CD73 influence self-renewal of E14Tg2A mESC **(A,B)**. Real-time PCR of the pluripotency markers OCT4 and NANOG in mESC cultured with or without the LIF under the inhibition of CD73 (α,β-methyleneadenosine 5′-diphosphate sodium salt) or ALPL (levamisole). GAPDH gene expression, which did not change under the used experimental conditions, was used as a reference. *p < 0,05. **(C)** BrdU-labeled cells were detected by flow cytometry after 96 hs of incubation with ALPL and CD73 inhibitors in the presence of the LIF. Cells without BrdU were used as the negative control for gating purposes, as demonstrated in the scatter plots. The average ± SE of three independent experiments is in the bar plot.

An important characteristic of ESCs is the capability of self-renewal. This capability ensures that cells during symmetric division during proliferation keep their pluripotency. In this regard, we challenged cells with levamisole (ALPL inhibitor) or α,β-MeADP (CD73 inhibitor) in the presence or absence of the LIF. Surprisingly, the inhibition of ALPL decreased 40% of both the levels of OCT4 and NANOG in LIF-treated cells (Figures 4A,B), hence highlighting its role in pluripotency maintenance. Following this, we tested if these enzymes would play any role in cell proliferation using BrdU quantification assay by flow cytometry. A common characteristic of undifferentiated ESC is that they skip the G1 checkpoint during the cell cycle, thus increasing the proliferation rates (Becker et al., 2006). Reinforcing the ALPL’s role in self-renewal, its inhibition led to decreased levels of proliferating cells reaching similar levels as that of the differentiated cells (around 42%) (Figure 4C), while undifferentiated cells presented over 60% of the population positive for BrdU.

Altogether, the data show that ALPL and ADA are important players in self-renewal signaling. ALPL starts the hydrolysis of ATP to adenosine. Adenosine deaminase (ADA) is important for the reduction levels of extracellular ADO levels. Once the levels of ADO increase, the cells compromise to differentiation, losing their pluripotency capacity.

### 3.2 Adenosine receptors and fate commitment

In light of our data showing extracellular levels of ADO as an important control of cell fate destiny, we investigated the role of the ADO receptors based on the determination of the levels of A1, A2A, A2B, and A3 subtype gene expression levels in undifferentiated and differentiated cells.

**FIGURE 5:**
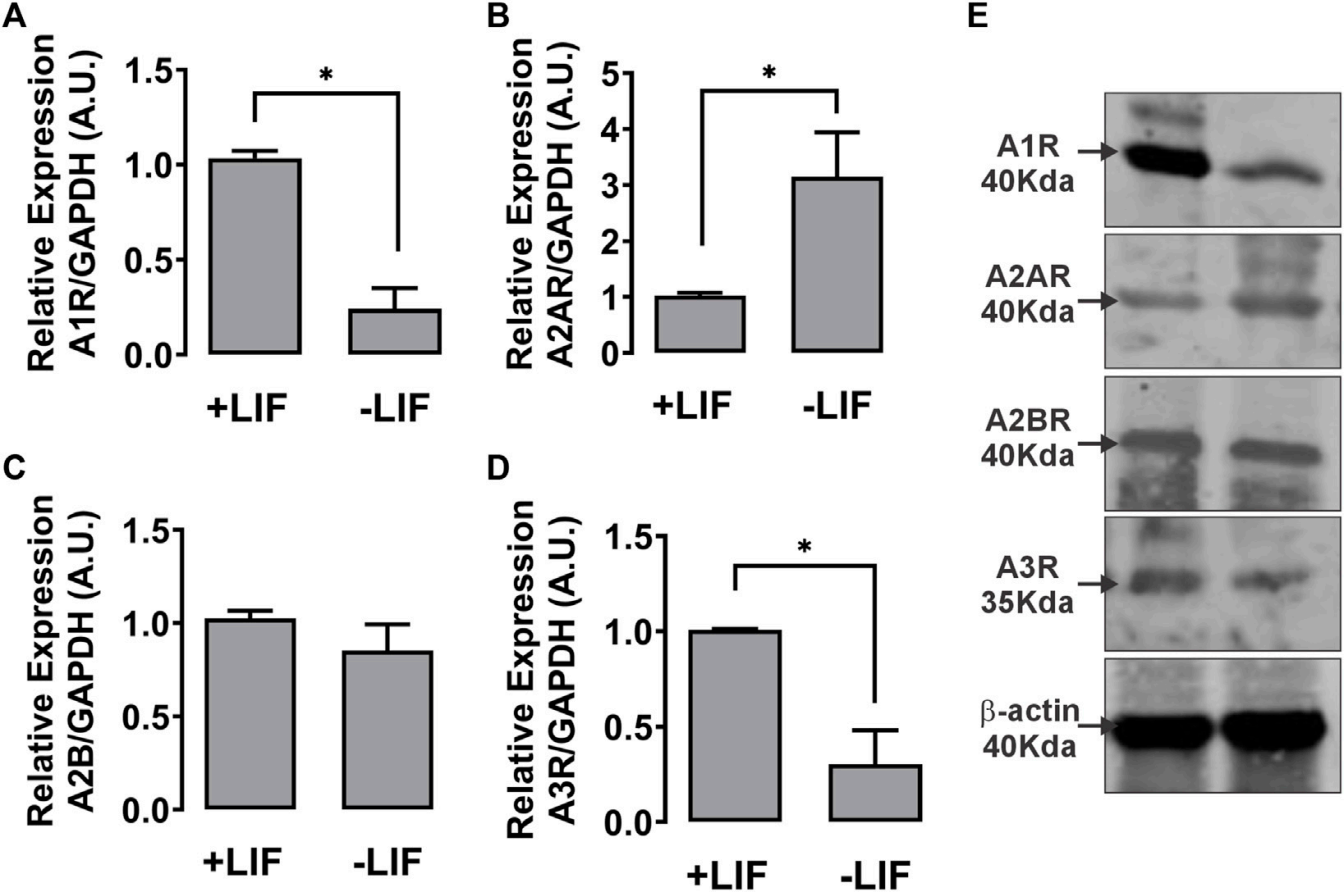
Expression of P1 receptor subtypes in E14Tg2A mESC. Real-time PCR of the four P1 subtypes showing the difference in the expression levels of adenosine A1 (A1R) **(A)**, A2A **(B)**, A2B **(C)**, and A3 **(D)** in mESCs cultured with or without the LIF. GAPDH gene expression, which did not change under the used experimental conditions, was used as a reference. **(E)** Western blot assay of mESC lysates for the detection of A1, A2A, A2B, and A3 receptors. Actin was used as the loading reference control. One-way ANOVA was conducted; *p ≤ 0,05. The experiments were independently repeated at least three times in triplicates.

As shown in Figure 5, the ESC culture under 96 h LIF deprivation showed decreased A1 and A3 receptor expression levels but increased A2A receptor levels when compared to LIF-treated cells.

**FIGURE 6:**
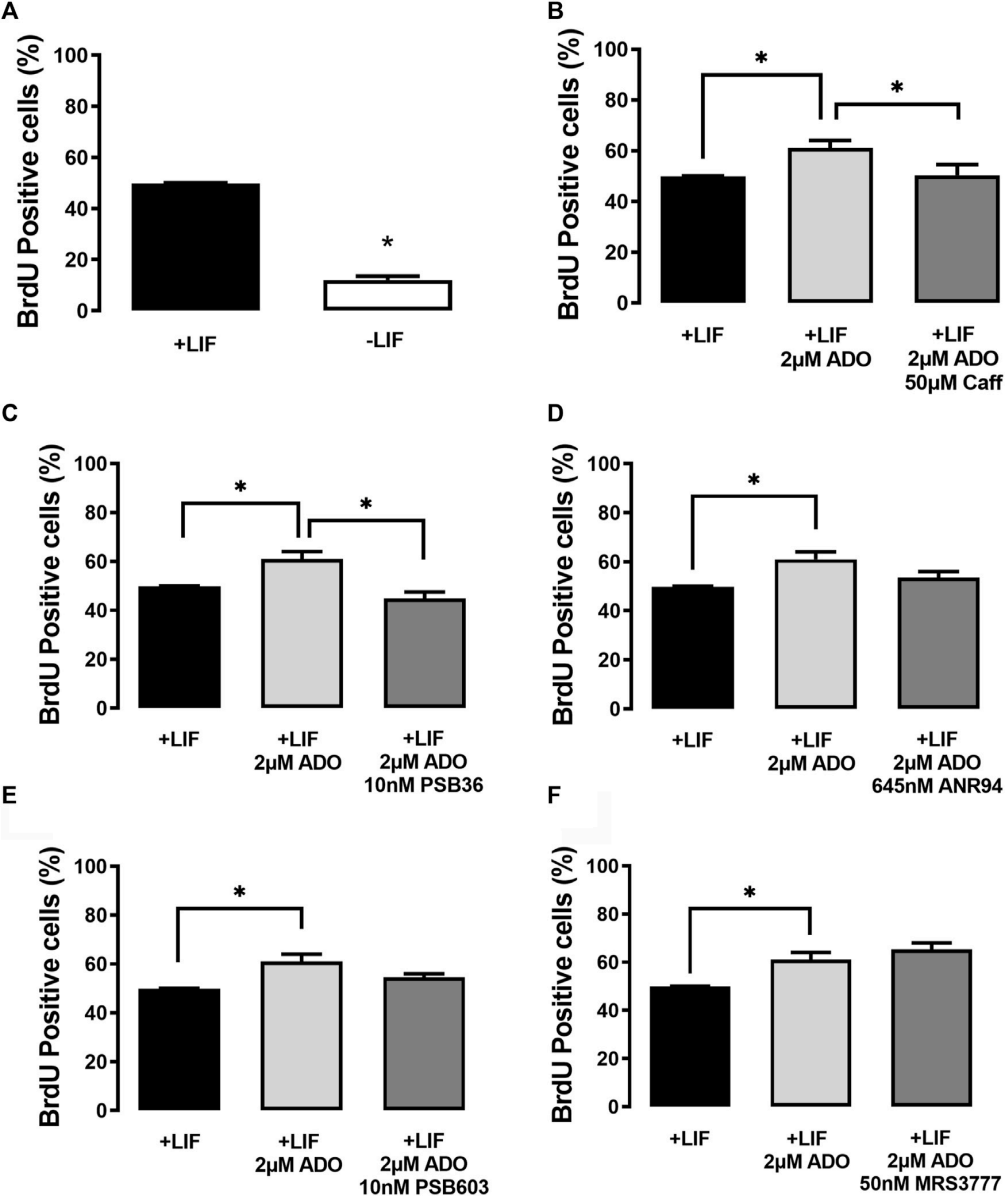
P1 receptor functions in the cell proliferation of undifferentiated E14Tg2A mESCs. BrdU labeling of ESC populations was determined by flow cytometry: **(A)** in the absence and presence of the LIF (undifferentiated), **(B)** in the presence of the LIF with 2 µM adenosine and with or without 2 µM adenosine and 50 µM caffeine (caff), and **(C–F)** with the LIF and adenosine in the presence and absence of the **(C)** A1 receptor antagonist receptor PSB36, **(D)** the A2A receptor antagonist ANR94, and **(E)** the A2B receptor antagonist PSB603 and the A3 receptor antagonist MRS3777. One-way ANOVA was conducted with the Bonferroni post-test; *p ≤ 0.05. The experiments were independently repeated at least three times in triplicates.

In the presence of the LIF (undifferentiated cells), ADO (2 µM) induced an increase of the percentage of BrdU-positive cells (Figure 6). This proliferative response is prevented by A1 receptor antagonism with PSB36 (10 nM). The A1 receptor expression is suppressed in undifferentiated cells cultured in the absence of the LIF, and, as such, these cells are less proliferative. The antagonism of receptors A2A with ANR94 (645 nM), A2B with PSB603 (10 nM), and A3 with MRS3777 (50 nM) did not evoke any proliferative effect (Figure 6).

**FIGURE 7:**
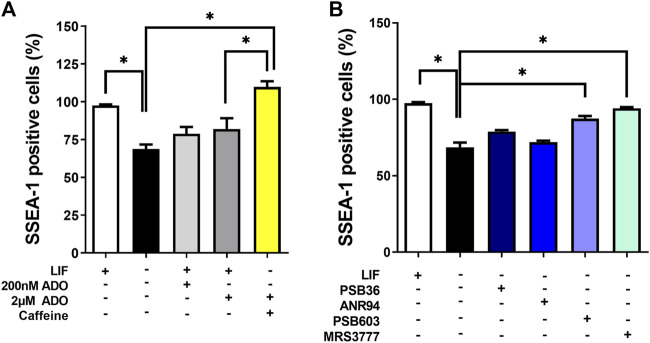
Functions of P1 receptors in the induction of spontaneous differentiation. The percentages of cells positive for SSEA-1 expression (mouse pluripotency marker) in the presence and absence of LIF in conditions of **(A)** inhibition by caffeine and/or stimulation by adenosine and **(B)** PSB36, ANR94, PSB603, and MRS3777, as A1, A2A, A2B, and A3 receptor subtype-selective antagonists for 96 h, respectively. One-way ANOVA was conducted with the Bonferroni post-test; *p ≤ 0.05. The experiments were independently repeated at least three times in triplicates.

Lower and higher doses of ADO in the culture could trigger different responses in OCT4 expression (Supplementary Figure S1). In addition to the transcription factors OCT4 and NANOG, SSEA-1 is another marker of pluripotency in murine ESC. Thus, the absence of the LIF matches with decreased SSEA1 expression levels, meaning that the cells are induced to differentiate. These levels are, however, restored even in the absence of the LIF when P1 blockers targeting either receptor A3 or A2B are applied (Figure 7).

**FIGURE 8:**
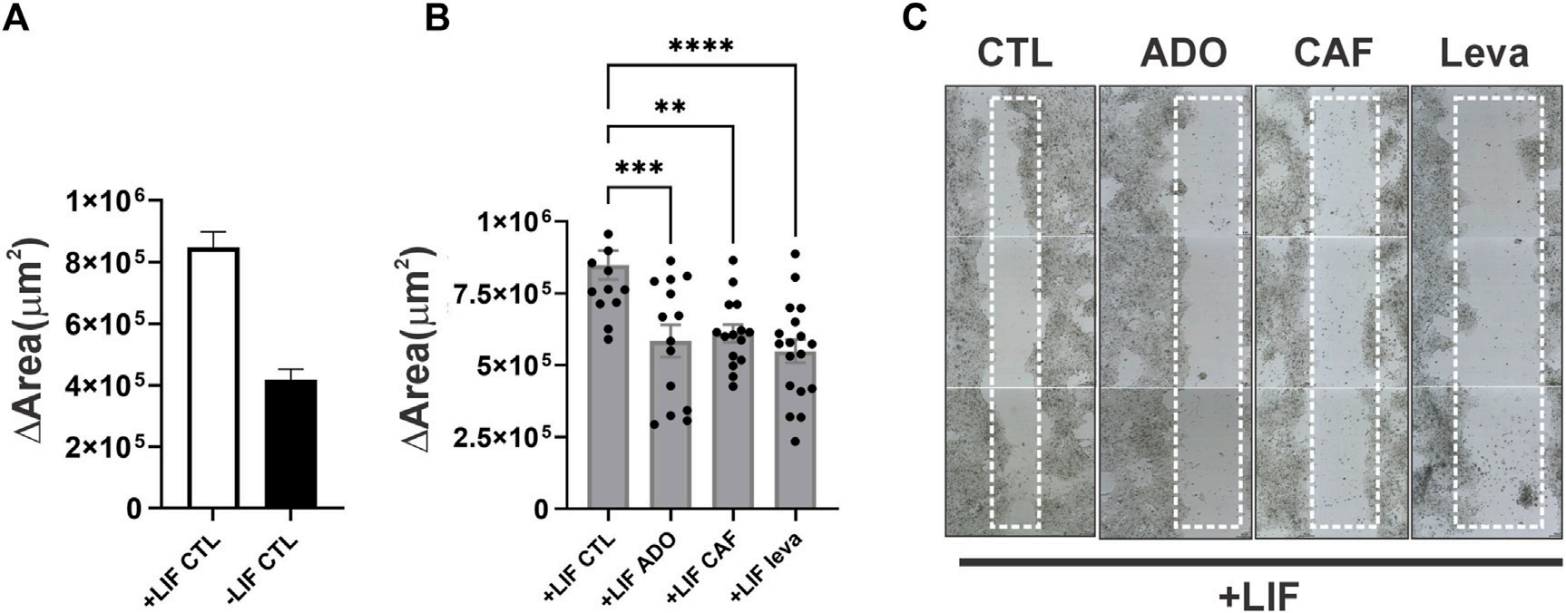
ADO signaling in the migration of undifferentiated and spontaneous differentiated E14Tg2A mESC **(A)**. Wound healing assay of cells cultured for 96 hs in the presence or absence of the LIF **(B)**. Some conditions had ADO (2 μM), caffeine (50 μM), or levamisole (1 mM, leva) applied for 96 hs **(C)**. Wells were completely scanned by TissueFAXS at 24 hs culture, and immediately after the scratch (0 h) and at 96 h (72 h after scratch), the quantification of the area was measured by tissue cytometry. The data plotted are the average ± SE of the variation of area (area72hs - area0h). One-way ANOVA; *p ≤ 0.05, **p ≤ 0.01, ***p ≤ 0.001. The experiments were independently repeated at least three times in triplicates.

In undifferentiated cells (the presence of the LIF), adenosine stimuli produce a slight decrease, although not statistically significant, in SSEA1 expression levels, suggesting a possible moderate shift towards cell commitment to differentiation. This decrease in SSEA-1 expression was reversed by A3 receptor inhibition, suggesting a role for this receptor in inducing cell differentiation. Cell migration was also important in differentiation (Figure 7). We show here through an *in vitro* wound healing assay that cells in the absence of the LIF migrate twice less than undifferentiated cells do and that both caffeine and levamisole treatment could induce cells under LIF treatment to migrate less, similar to the removal of the LIF (Figure 8).

**FIGURE 9:**
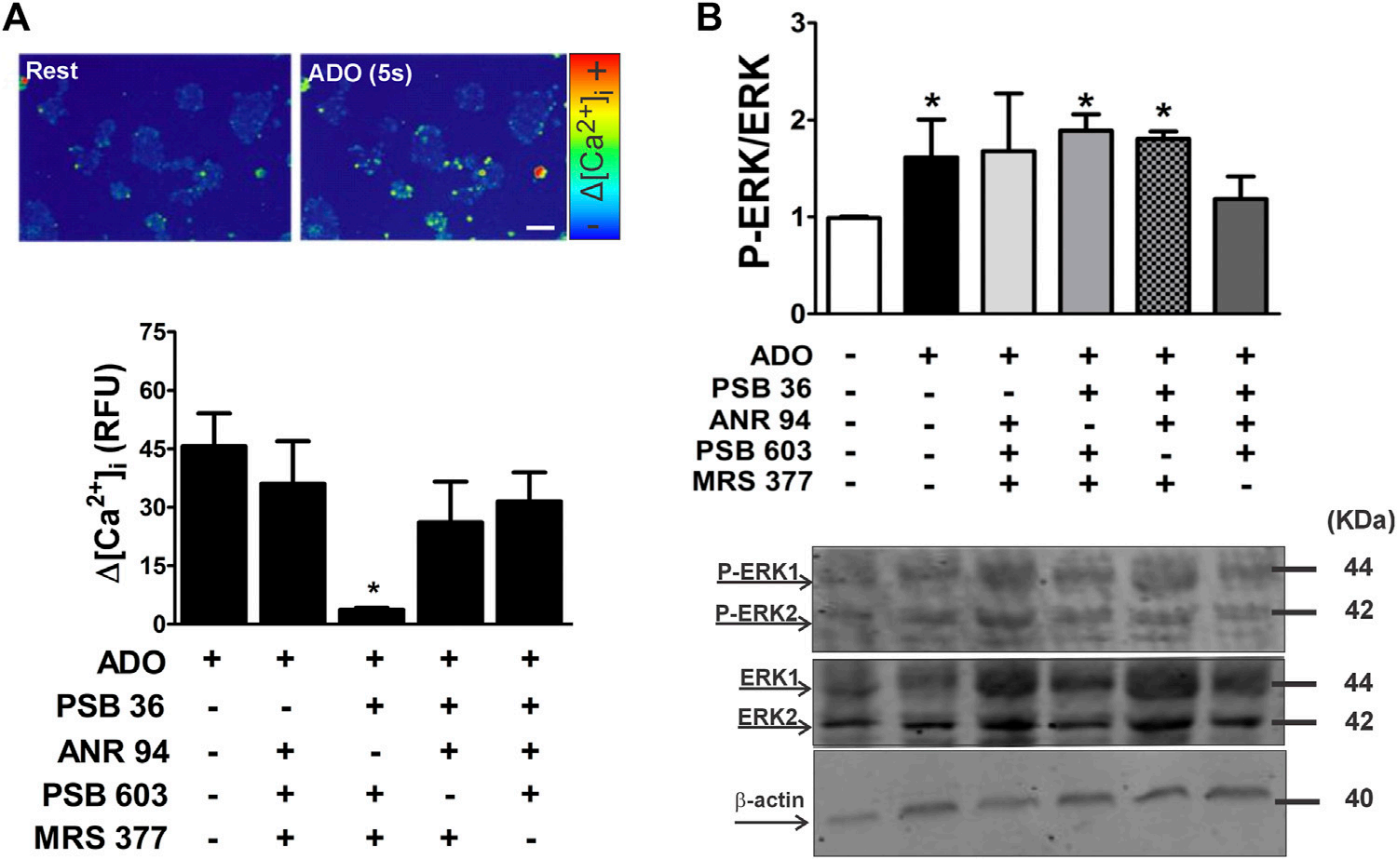
Adenosine receptor subtype-selective [Ca2+]i mobilization and ERK phosphorylation in undifferentiated ESC using antagonist cocktails. **(A)** Upper panel: [Ca^2+^]_i_ mobilization was measured in resting conditions (rest) and in the presence of adenosine following staining of mESCs with fluo3-AM to assess responses on the single-cell level. Lower panel: adenosine-induced [Ca^2+^]_i_ transients were determined using microfluorimetry in the absence and presence of adenosine and A1, A2A, A2B, and A3 receptor subtype-selective antagonists. **(B)** ERK1/2 protein expression levels in undifferentiated cells. Phosphorylated ERK (pERK) and total ERK protein expression levels in ESCs treated for 10 min with different concentrations of adenosine. Quantification of pERK/ERK in the absence and presence of A1, A2A, A2B, and A3 receptor subtype-selective antagonists was carried out after 5 min of incubation. Protein extracts were separated by SDS-PAGE and submitted to Western blot analysis using ERK- and pERK-specific antibodies. One-way ANOVA was conducted. *p ≤ 0.05. P1 subtype antagonists: PSB36 = A1; ANR 94 = A2A; PSB603 = A2B; and MRS 377 = A3. The experiments were independently repeated at least three times in triplicates.

In addition, the stimulation of ESC with ADO (2 μM) triggered intracellular calcium response (Figure 9A). These data are pioneering, since the P1 receptors are not usually coupled to the Gq protein. In some cases, the A1 and A3 receptor can trigger calcium response by phospholipase C through the activation of a pertussis toxin-sensitive G protein in rat basophilic leukemia cells (Ali et al., 1991; Sheth et al., 2014) or by coupling to the Gq protein (Fredholm et al., 2011). The mitogen-activated protein kinase (MAPK) pathway can also be activated by the βγ subunit of the Gi/o protein (Faure et al., 1994). The activation of the A2A receptor usually triggers the Gs protein, which can lead to cyclic AMP production, thus indirectly activating protein kinase C (Sheth et al., 2014). Contrarily, the A2B receptor can stimulate PKC and ERK1/2 activity by directly coupling to Gq proteins (Sheth et al., 2014).

The inhibition of MAPK favors pluripotency maintenance in mESCs (Faure et al., 1994). In this regard, we checked if the activation of any of the ADO receptor subtypes could trigger ERK phosphorylation. The strategy consisted of using cocktails that leave just one receptor available to be activated by ADO. Under conditions of LIF deprivation for 10 min, adenosine stimulus increases phosphorylation of ERK 1/2, a change prevented by the antagonism of either A2A or A2B receptors (Figure 9B). ADO receptors can be coupled to Gq, Gi, or Gs proteins.

Intracellular calcium levels were rised upon stimulation with adenosine whenever A1, A2B, or A3 receptors are active (Figure 9A), and basal calcium levels are persistent when adenosine challenge occurs in the presence of a cocktail of inhibitors for these P1 receptors.

**FIGURE 10:**
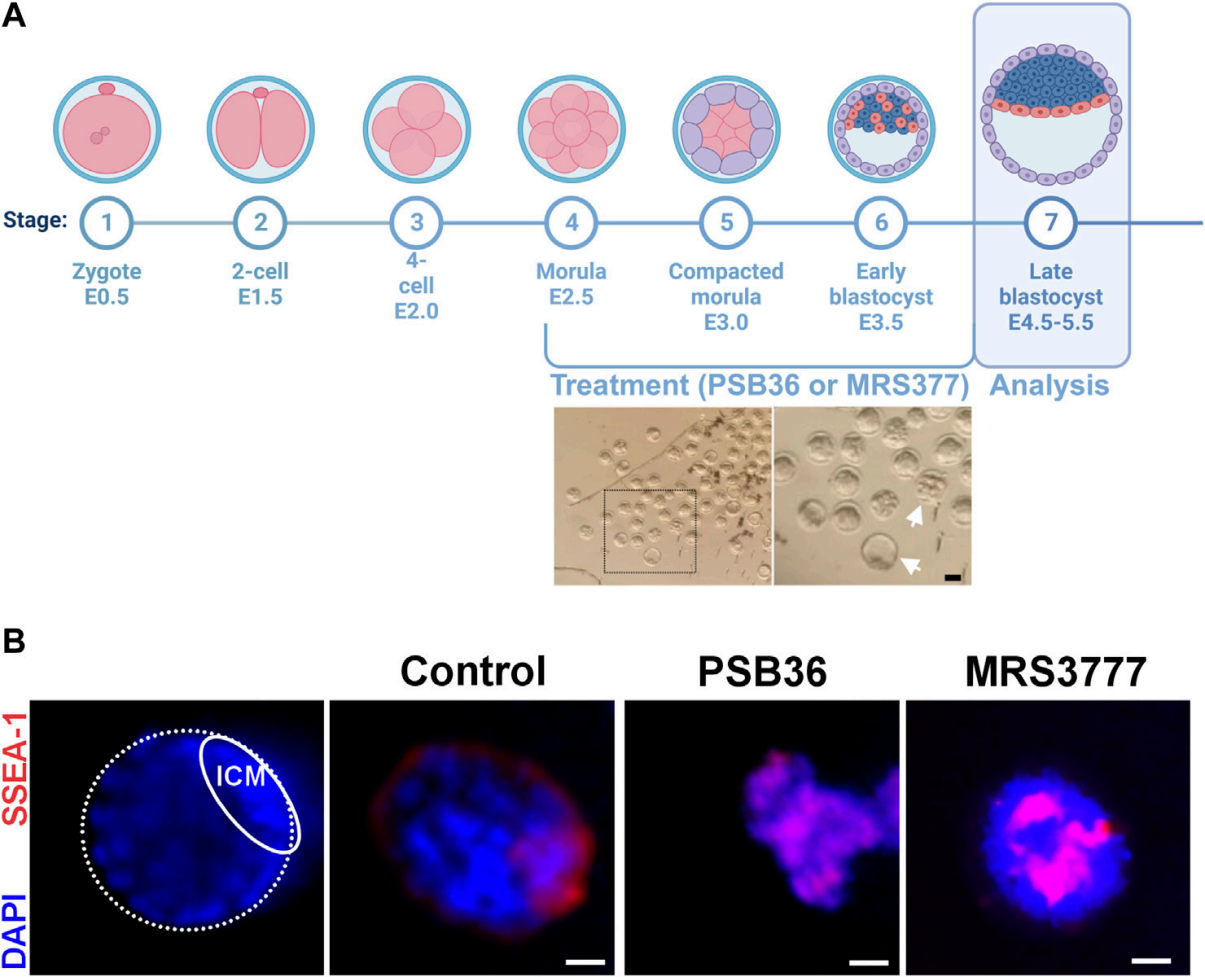
ADO receptor modulation during mouse embryo development. **(A)** Mouse embryos isolated 3.5 days after fecundation show different stages of development, as indicated by the white arrows in the photomicrography. Created with BioRender.com. **(B)** Immunostaining for SSEA-1 (pluripotency marker, red) and the nuclei (DAPI, blue) of mouse embryos treated for 2 days with 10 nM PSB36 (A1 receptor antagonist) or 50 nM MRS 377 (A3 receptor antagonist). There were a total of 15 embryos for each condition plus the experimental control. Scale bar: 10 μm. The experiments were independently repeated at least three times in triplicates.

In order to validate our findings in an *in vivo* model, we treated isolated mouse morula with PSB36 (A1 receptor antagonist) and MRS3777 (A3 antagonist) and waited until they reached the blastula stage (Figure 10A). The embryos treated with PSB36 showed smaller sizes and amorphous shapes, confirming our findings that the inhibition of the A1 receptor impairs proliferation and self-renewal. The embryos treated with MRS3777 were spherical and round with intense SSEA-1 staining in the core of the embryo, indicating disrupted differentiation, as we observed *in vitro* (Figure 10B).

## 4 Discussion

Our data indicate that ALPL and ADA favor lower concentration of extracellular ADO, controlling ESC proliferation through A1 receptor activation, triggering [Ca^2+^]_i_ transients and self-renewal. Calcium signaling has been related to proliferation and differentiation in many studies (Pinto et al., 2015). Induction of calcium signaling induced by A1 receptors has been shown before in human bronchial smooth muscle cells (Ethier and Madison, 2006), and Jones and collaborators used calcium flux measurements for the screening of adenosine receptor agonists (Jones et al., 2017).

On the other hand, CD73 favors high concentrations of extracellular ADO that binds to the A2B and A3 receptors, which have a lower affinity to ADO, triggering the MAP kinase pathway, leading to differentiation in mESC *in vitro* and *in vivo*. Therefore, the fine-tuning balance of extracellular ADO plays a crucial role in embryo development signaling. The pharmacological impairment of ADO receptors could trigger embryo development issues, which could lead to malformations or miscarriages. Caffeine is a commonly used drug that can impair every adenosine receptor.

Self-renewal is a very important characteristic of stem cells because it allows them to replicate symmetrically, keeping the same patterns as the mother cell. Differentiation is also important in the embryo development context; otherwise, the embryo would not form the various cell types of the fetus, and it is caused by asymmetrical cell division during mitosis. The constant increase of eADO allows A2B and A3 receptors to be activated and induces a pro-differentiation signaling. This claim is supported by the observation that an increase in eADO concentration favors proliferation, which is needed for asymmetrical division as well. As expected, pro-proliferation and differentiation eADO signaling was blocked in the presence of caffeine, which blocked this pro-signal. These data showed that LIF signaling has a feedback loop with P1 receptors, and caffeine could block this signaling, even without the LIF, demonstrating that P1 receptors are hierarchically upstream in this feedback control.

Caffeine has been shown before to impair stem cell proliferation and differentiation. For instance, the N-ethylcarboxamide-induced migration and proliferation of mouse embryonic stem cells was blocked by caffeine (Kim et al., 2010). In line, Houghton et al. (2020) showed that caffeine exposure affected the proliferation of hippocampal neural progenitor cells. Furthermore, caffeine negatively affected the osteogenic differentiation of mesenchymal stem cells derived from the offspring of rats during pregnancy and lactation (Reis et al., 2016).

Prenatal caffeine exposure was studied in animal models. Effects of daily caffeine exposure in mice, such as an equivalent of three coffee cups during pregnancy in humans, showed alterations in the GABAergic neuron networks and augmented synaptic activity in the primary visual cortex *in vitro* and *in vivo*. Such enhanced synaptic activity made animals prone to seizure activity (Fazeli et al., 2017). Adverse effects are related to adenosine receptor inhibition. The A1 receptors are expressed early in neural development and are neuroprotective (Rivkees, 1995). Overall, as reviewed by Rivkees and Wendler (2011), ADO-promoted neuroprotection in the embryo by caffeine can also augment miscarriage and fetal growth retardation risks. In addition to extensive *in vitro* and *in vivo* studies with rodent models, data from human studies are also available. An adolescent brain and cognitive development study with valid data for prenatal caffeine exposure revealed structural alterations in the brain such as increased posterior and lower frontal cortical thickness and changes in the parieto-occipital sulcal depth (Zhang et al., 2022).

A multi-center study with 2,635 low-risk pregnant women recruited subjects between 8–12 weeks of pregnancy and observed that caffeine consumption carries the risk of fetal growth restriction, which may be related to caffeine-induced proliferation inhibition (CARE Study Group, 2008). The authors of this work observed that daily consumption of 200 mg of caffeine resulted in a lesser birth weight of 60–70 g (*p* = 0.004). The loss of birth weight tended to increase with higher daily caffeine consumption. Previous studies also showed similar results, emphasizing a relationship between caffeine and growth inhibition (Chen et al., 2014).

Steroid hormone-regulated gene dysregulation, such as the LIF, mucin, lactoferrin, and amphiregulin, are crucial determinants of uterine receptivity. Thus, they could be detected in the preimplantation uterine epithelium after caffeine treatment, compromising the nidation (Qian et al., 2020). Altogether, the data show that caffeine influence is prejudicial for embryo development. Herein, we scavenged the mechanism by which caffeine action modulates ADO receptors during the early embryo development. Therefore, pregnancy complications and pregnancy loss occur.

## 5 Conclusion

Essentially, ALPL and ADA are highly expressed in mESCs and participate in the maintenance of ESC pluripotency and proliferation (self-renewal) by maintaining lower levels of ADO in the extracellular milieu. Inhibiting ALPL activity is sufficient to differentiate these cells even in the presence of the LIF, which is known to help in maintaining pluripotency. Since both enzymes manage the balance of extracellular ADO availability, consequently, they modulate the activity of the P1 receptor subtypes. In this case, the A1 receptor subtype, which is highly expressed in undifferentiated cells, transmits the signal for self-renewal by calcium transients.

Moreover, the CD73 enzyme produces higher levels of extracellular ADO, leading to differentiation by triggering the A2B and A3 receptors through the modulation of calcium and MAPK/ERK signaling pathways. The data are also valid for the embryo model, indicating that ADO antagonism by caffeine exposure can lead to disrupted development.

## Data Availability

The raw data supporting the conclusion of this article will be made available by the authors, upon reasonable request.
