# Supplementary figures and images for: Impairment of adenosine signaling disrupts early embryo development: unveiling the underlying mechanisms
Source: Front Pharmacol. 2024 Jan 19;14:1328398. doi: 10.3389/fphar.2023.1328398 (PMC10834787; doi:10.3389/fphar.2023.1328398)

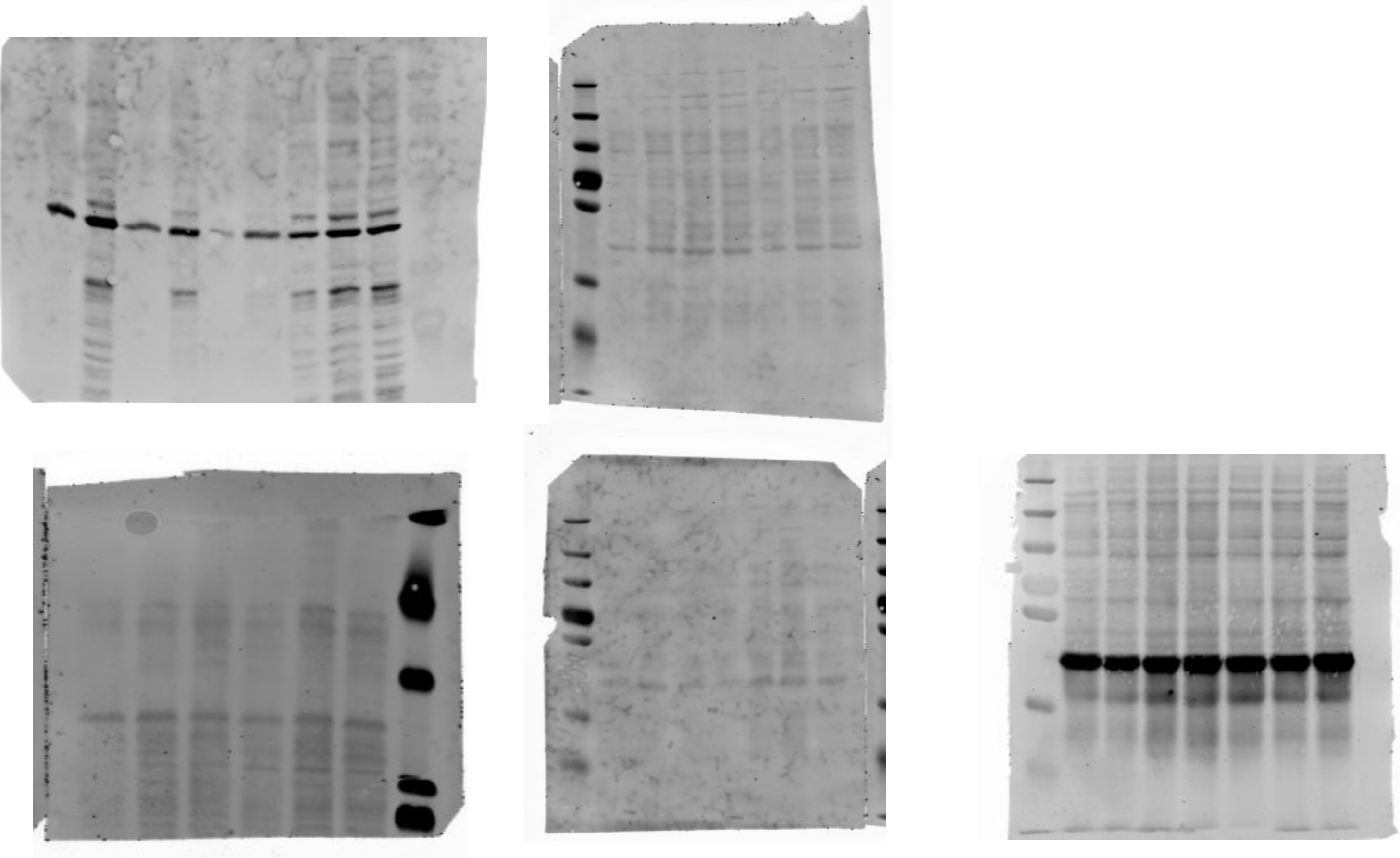

Supplement: Supplementary file 1 [file Image3.TIF]

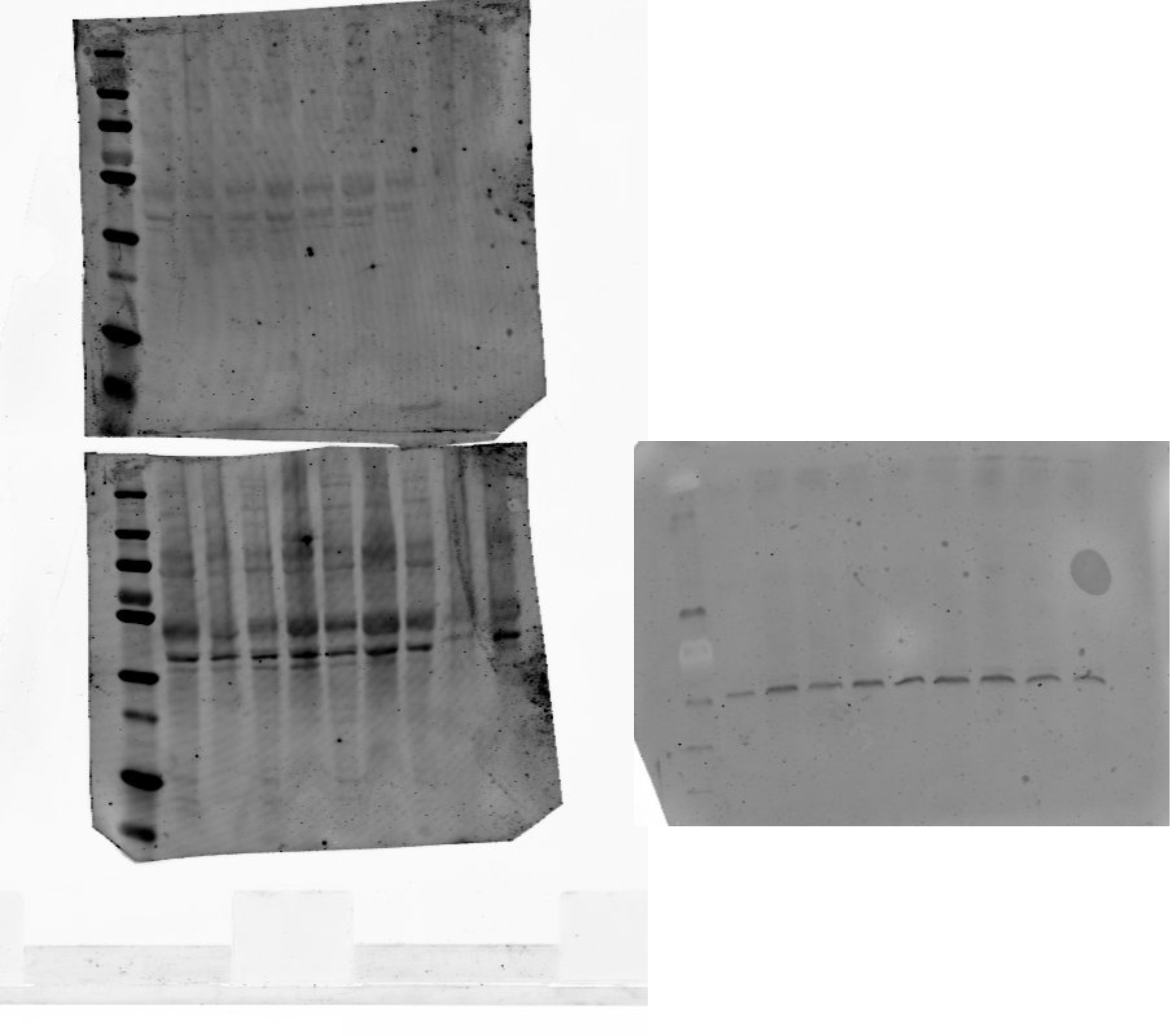

Supplement: Supplementary file 2 [file Image4.TIF]

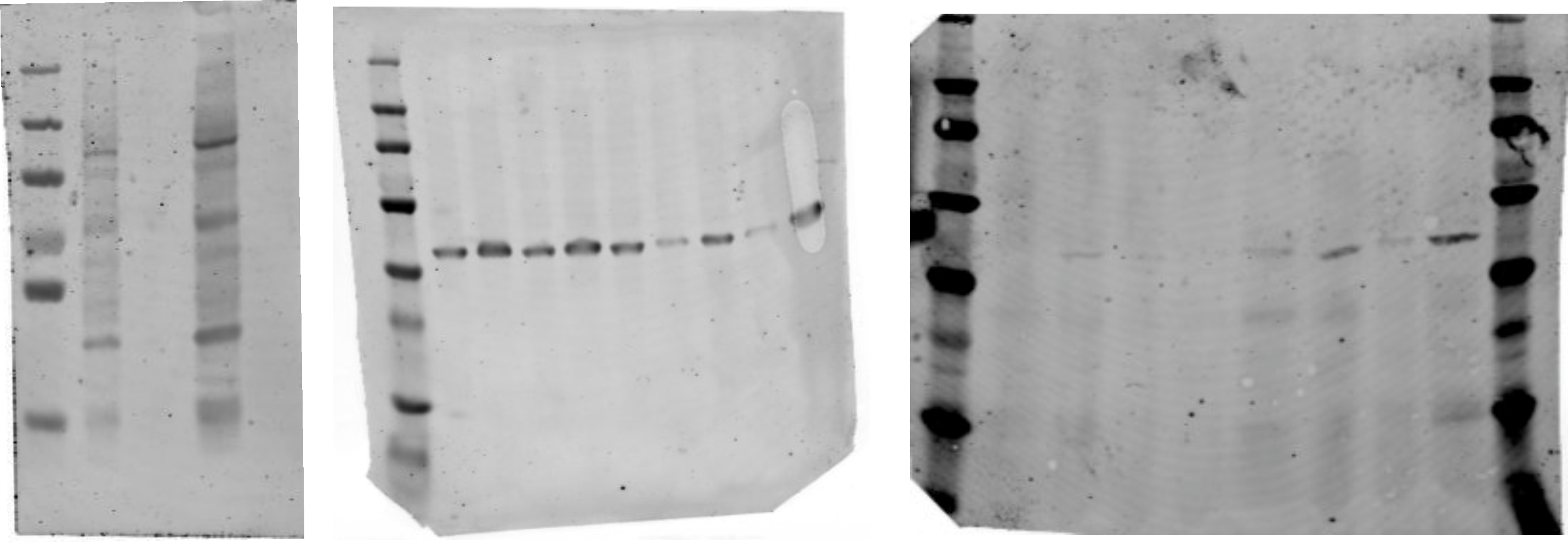

Supplement: Supplementary file 3 [file Image2.TIF]

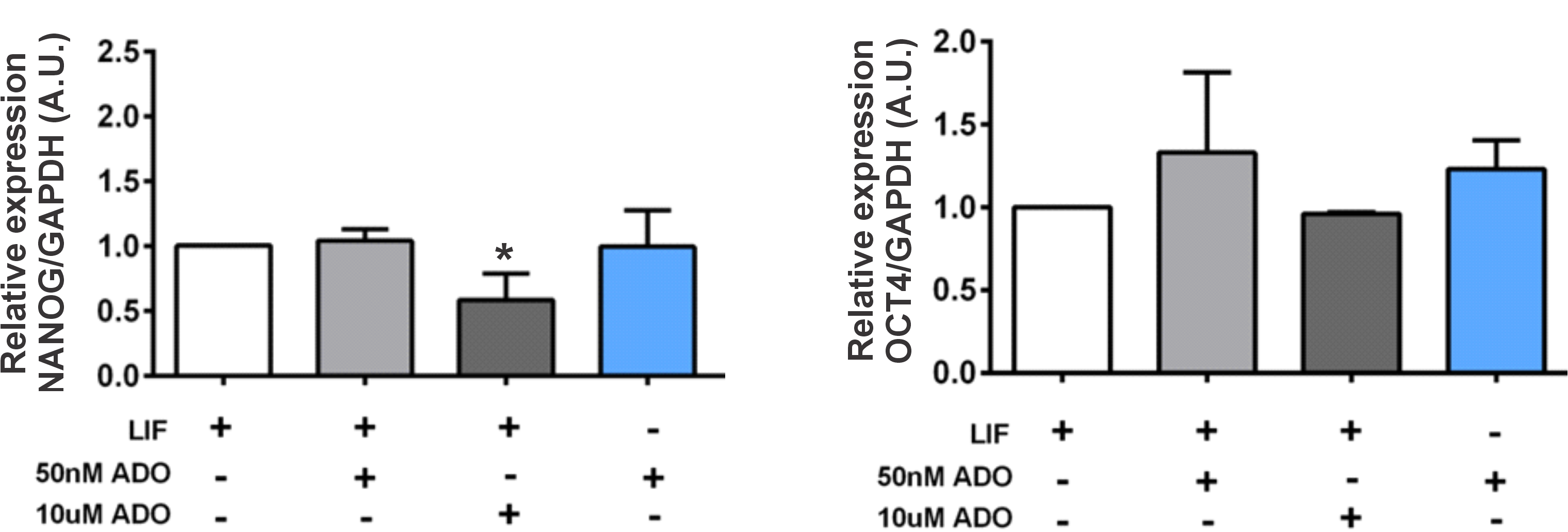

Supplement: Supplementary file 4 [file Image1.TIF]
